# RETRACTION: Effect of Hyperbaric Oxygen Treatment on Diabetic Foot Ulcers: A Meta‐Analysis

**DOI:** 10.1111/iwj.70267

**Published:** 2025-02-25

## Abstract

**RETRACTION:** ImamM. S. , AlmutairiA. K. , AlhajriA. M. , AlharbyM. M. , AlanaziM. H. , AlotaibiA. G. , and AbdelrahimM. E. A. , “Effect of Hyperbaric Oxygen Treatment on Diabetic Foot Ulcers: A Meta‐Analysis,” International Wound Journal 21, no. 2 (2024): e14427, 10.1111/iwj.14427.PMC1082872837795772

The above article, published online on 05 October 2023, in Wiley Online Library (http://onlinelibrary.wiley.com/), has been retracted by agreement between the journal Editor in Chief, Professor Keith Harding; and John Wiley & Sons Ltd. Following an investigation by the publisher, all parties have concluded that this article was accepted solely on the basis of a compromised peer review process. The editors have therefore decided to retract the article. The authors did not respond to our notice regarding the retraction.

**RETRACTION:** M. S. Imam , A. K. Almutairi , A. M. Alhajri , M. M. Alharby , M. H. Alanazi , A. G. Alotaibi , and M. E. A. Abdelrahim , “Effect of Hyperbaric Oxygen Treatment on Diabetic Foot Ulcers: A Meta‐Analysis,” International Wound Journal 21, no. 2 (2024): e14427, 10.1111/iwj.14427.PMC1082872837795772

The above article, published online on 05 October 2023, in Wiley Online Library (http://onlinelibrary.wiley.com/), has been retracted by agreement between the journal Editor in Chief, Professor Keith Harding; and John Wiley & Sons Ltd. Following an investigation by the publisher, all parties have concluded that this article was accepted solely on the basis of a compromised peer review process. The editors have therefore decided to retract the article. The authors did not respond to our notice regarding the retraction.

